# Supplementary material for: WordCommentsAnalyzer: A windows software tool for qualitative research
Source: F1000Res. 2018 Sep 4;7:536. Originally published 2018 May 3. [Version 2] doi: 10.12688/f1000research.14819.2 (PMC6137411; doi:10.12688/f1000research.14819.2)
Supplement: Supplementary file 3 [file f1000research-7-17614-s0002.tgz › b2a79496-4ada-47c4-9682-a99ce867836d.zip!/Eight Iranian Health Journals/Archives of Rehabilitation_43.docx]

© Archives of Rehabilitation. This work is licensed under CC BY-NC 4.0 (https://creativecommons.org/licenses/by-nc/4.0/).

Volume & Issue: دوره 10 - شماره 1

Title: گزارش یک مورد بیمار فارسی‌زبان مبتلا به نشانگان لهجه خارجی

http://rehabilitationj.uswr.ac.ir/article-1-315-fa.html

نشانگان لهجه خارجی (FAS) اختلال گفتاری کمیابی است که در اثر ضایعات وارده بر نیمکره چپ مغز ایجاد می‌گردد. در این نشانگان عموماً ترکیب انحرافات زنجیری و زبرزنجیری در تولید سبب ایجاد لهجه خارجی می گردد. جدا از مشکلات تلفظی زنجیری، خطاهای زبرزنجیری غالباً مختصه اصلی نشانگان لهجه خارجی در نظر گرفته می‌شوند. در این مقاله گزارشی از یک زن فارسی زبان 53 ساله ارائه می‌شود که پس از سکته مغزی و وقوع انفارکتوس‌های کانونی در مرکز نیمه بیضی نیمکره چپ، با لهجه خارجی صحبت می‌کند. بر اساس اطلاعات محققان و پیگیری‌های صورت گرفته، احتمالاً این بیمار اولین مورد نشانگان لهجه خارجی گزارش شده در ایران می‌باشد. ویژگی‌ها‌ی زبرزنجیری گفتار این بیمار در مقایسه با مختصه‌های گفتاری سخنگویان طبیعی زبان فارسی و با استفاده از نرم‌افزار‌Praat نسخه ۴-۱-۹ مورد بررسی قرارگرفته است. نتایج به‌دست آمده در راستای یافته‌های تحقیقات پیشین و نشانگر آن هستند که انحرافات عمده در ویژگی‌های زبرزنجیری گفتار این بیمار در ارتباط با تکیه، سرعت گفتار و درنگ رخ می‌دهند.

Title: تأثیرات فیزیولوژیک تمرینات بدنی مقاومتی و استقامتی بر بیماران مبتلا به بیماری‌‌های قلبی-عروقی

http://rehabilitationj.uswr.ac.ir/article-1-314-fa.html

این مقاله مروری، ارائه شواهد علمی در خصوص آنکه چگونه تمرینات مقاومتی و هوازی کلید اصلی سلامتی، آمادگی جسمانی و طول عمر است و دلایلی که چرا هر دو نوع تمرین موجود در برنامه فعالیت جسمانی جهت افزایش ظرفیت تمرینی بیماران قلبی- عروقی مناسب است، می‌باشد. هم اکنون بیماری‌‌های قلبی– عروقی از دلایل اصلی مرگ و میر و بیماریزایی در سراسر دنیا هستند. هر چند که عوامل ژنتیکی و سن از عوامل تعیین‌کننده مهم و خطر ساز این بیماریها هستند، لیکن سایر عوامل شامل فشار خون، افزایش کلسترول خون، مقاومت در برابر انسولین، دیابت و مشکلات موجود در شیوه زندگی نظیر سیگار کشیدن و رژیم غذایی نیز جزء عوامل خطر ساز مرتبط با بیماری محسوب می‌شوند. پژوهش‌‌ها نشان می‌دهند که نوتوانی قلب در کاهش خطر مرگ پس از سکته قلبی مهم بوده و تجویز انواع تمرینات موجب بهبود حداکثر اکسیژن مصرفی می‌گردد و احتمالاً کیفیت زندگی بیماران قلبی- عروقی را بهبود می‌بخشد. یافته‌‌ها بیانگر آن است که بیماران عروق کرونر دچار کاهش حداکثر توان هوازی، قدرت عضلانی، کیفیت زندگی و فعالیت بدنی هستند که منجر به افزایش خطر شیوع عوامل خطر ساز این بیماری می‌شود که با افزایش سن افزایش می‌یابند. تمرینات بدنی موجب افزایش حداکثر اکسیژن مصرفی و قدرت عضلانی شده و ممکن است موجب بهبود کیفیت زندگی شوند. امروزه تمرینات بدنی جزء مهم درمان بیماری عروق کرونری محسوب می‌شود که موجب افزایش ظرفیت عملکردی، کیفیت زندگی و پیش آگهی بیماری می‌گردد و به‌طور روز افزون در برنامه‌های درمانی این بیماران مورد استفاده قرار می‌گیرد.

Title: سفری همراه والدین کودکان ناتوان: از تشخیص تا تطابق

http://rehabilitationj.uswr.ac.ir/article-1-313-fa.html

هدف: این مطالعه به بررسی تجربیات، انتظارات و نیازهای والدین کودکان ناتوان، در ارتباطشان با ارائه‌دهندگان خدمات تخصصی و همچنین نقش این افراد در روند تطابق والدین با ناتوانی فرزندشان می‌پردازد. روش بررسی: این مطالعه به صورت کیفی و به شیوه پدیدار شناسانه انجام گرفته است. والدین 9 کودک مبتلا به ناتوانی‌های مختلف، به صورت هدفمند انتخاب شده و مورد مصاحبه نیمه ساختار یافته عمیق قرار گرفتند. مصاحبه‌ها ضبط شده و سپس به‌صورت کتبی پیاده شد. داده‌های حاصل با استفاده از روش ون مانن تجزیه و تحلیل شد. یافته‌ها: تجربیات والدین در رابطه با خدمات تخصصی ارائه شده به فرزندان ناتوان‌ آنها در چهار حوزه قابل طرح و دسته‌بندی است. این چهار حوزه عبارتند از: خدمات پزشکی، مشاوره، توانبخشی و آموزشی. در حوزه خدمات پزشکی، مهارت برقراری ارتباط، تشخیص به‌موقع و بیان واقعیت نمود داشت. خدمات مشاوره مشتمل بر مشاوره حمایتی و آموزشی بود. خدمات توانبخشی، پیشرفت عملکردی کودک، امکان مشاهده و مقایسه دیگر کودکان با شرایط مشابه و نگرش درمانگران را در بر داشت. خدمات آموزشی شامل آموزش کنونی و آینده می‌گردید. نتیجه‌گیری: نتایج این مطالعه بر مسائل و چالشهای مختلفی که والدین در زمینه برخورداری از خدمات با آنها مواجه هستند، دلالت دارد. این نتایج نشان می‌دهد که با تشخیص و مداخله به‌موقع، ارتباط همدلانه و مشاوره سودمند، می‌توان به والدین در تطابق و کنار آمدن با معلولیت فرزندشان کمک کرد تا بتوانند با ایجاد یک نگرش مثبت به مراقبت و پرورش کودکشان بپردازند.

Title: طراحی و ساخت اسپلینت جدید عنکبوتی دست و مقایسه تأثیر آن بر بهبودی عملکرد دست با اسپلینت فلزی معمول

http://rehabilitationj.uswr.ac.ir/article-1-311-fa.html

هدف: این پژوهش با هدف طراحی و ساخت نوع جدیدی از اسپلینت عنکبوتی و مقایسه تأثیر آن با اسپلینت فلزی معمول بر بهبود عملکرد دست بیماران دارای آسیب عصب رادیال انجام شد. روش بررسی: در این مطالعه شبه‌تجربی از بیماران دارای آسیب عصب رادیال که به درمانگاه بیمارستان‌های سینا و امام خمینی تهران مراجعه کرده بودند، ۲۴ نفر به طور تصادفی انتخاب و پس از تخصیص تصادفی تعادلی به دو گروه، تحت درمان به دو روش استفاده از اسپلینت جدید و اسپلینت فلزی معمول قرار گرفتند. میزان دامنه حرکات مفصلی مفاصل مچ، کف‌دستی-انگشتی، بین‌انگشتی‌نزدیک، بین‌انگشتی‌دور و همچنین میزان راحتی و اگزمای پوستی بیماران قبل از مداخله و 4 ماه پس از استفاده از اسپلینت‌ها ارزیابی و نتایج حاصل با استفاده از آزمون‌های آماری مجذور خی و تی‌مستقل مورد تحلیل قرار گرفت. یافته‌ها: میانگین دامنه حرکتی مفاصل مچ (۰/۰۰۱>P)، کف‌دستی-انگشتی (۰/۰۰۳=P) و بین‌انگشتی‌نزدیک (۰/۰۲۹=P) بین دو گروه تفاوت معناداری داشت، ولی دامنه حرکتی مفصل بین‌انگشتی‌دور دو گروه تفاوت معناداری نداشت (۰/۰۵۴=P)، اگرچه نتایج بهبودی گروه اسپلینت عنکبوتی در این مفصل نیز بهتر از گروه دیگر بود. نتیجه‌گیری: اسپلینت جدید طراحی شده و اسپلینت فلزی معمول هر دو در بهبودی عوارض ناشی از آسیب عصب رادیال (عدم اکستنشن انگشتان، افتادگی مچ و بدشکلی ثانویه) مؤثر می‌باشند، ولی اسپلینت جدید به مراتب بهتر از اسپلینت قدیمی عملکرد دست را بهبود می‌بخشد.

Title: مقایسه مشکلات رفتاری و پیشرفت تحصیلی در کودکان با و بدون مشکلات حرکتی

http://rehabilitationj.uswr.ac.ir/article-1-310-fa.html

هدف: این پژوهش به‌منظور مقایسه مشکلات رفتاری و پیشرفت تحصیلی در کودکان با و بدون مشکلات حرکتی انجام گرفت. روش بررسی: در این تحقیق مقایسه‌ای هفده کودک با مشکلات حرکتی و بیست و پنج کودک بدون مشکلات حرکتی که مهارتهای حرکتی آنان بر اساس مقیاس رشد حرکتی لینکلن- اوزرتسکی سنجیده شده بود، به‌طور تصادفی انتخاب و وضعیت رفتاری و تحصیلی آنها مورد مقایسه قرار گرفت. از فرم والد چک لیست رفتاری کودک برای ارزیابی مشکلات هیجانی و رفتاری کودکان استفاده شد. داده‌های حاصل با استفاده از آزمون تی‌مستقل تحلیل شدند. یافته‌ها: نتایج نشان داد کودکان دچار مشکلات حرکتی در مقایسه با کودکان بدون مشکلات حرکتی پیشرفت تحصیلی ضعیف تری دارند (P<0/001). همچنین نتایج نشان داد که کودکان دارای مشکلات حرکتی، مشکلات کلی، درونی، بیرونی و اجتماعی، شکایات جسمانی، عدم توجه و گوشه‌گیری بیشتری نسبت به کودکان بدون مشکلات حرکتی دارند (۰/۰۰۱>P)، ولی بین دو گروه از نظر اضطراب/ افسردگی، مشکلات تفکر و جنسیت و رفتار بزهکارانه و پرخانشگرانه اختلاف معناداری وجود ندارد (P<0/05). نتیجه‌گیری: مطابق نتایج حاصل، کودکان دارای مشکلات حرکتی خطرپذیری بیشتری برای ابتلا به مشکلات در زمینه‌های رفتاری و تحصیلی دارند. ازاین‌رو در ارزیابی این کودکان (صرف‌نظر از میزان یا شدت این مشکلات) می‌بایست دامنه وسیعی از کنش‌های آنان علاوه‌بر کنش حرکتی مورد بررسی قرار گیرد.

Title: تأثیر مداخلات به‌هنگام روان‌شناختی و آموزشی خانواده‌محور

http://rehabilitationj.uswr.ac.ir/article-1-309-fa.html

هدف: پژوهش حاضر با هدف بررسی تأثیر مداخلات به‌هنگام روان‌شناختی و آموزشی خانواده محور بر رشد حرکتی کودکان با نشانگان داون زیر 5 سال در شهر اصفهان انجام گرفت. روش بررسی: در این پژوهش تجربی و مداخله‌ای، والدین 36 کودک مبتلا به نشانگان داون که به‌صورت تصادفی ساده انتخاب و به‌طور تصادفی تعادلی در دو گروه مداخله و کنترل قرار گرفتند، با استفاده از مدل مشاوره‌‌ای «هورن بی» تحت مداخلات روان‌شناختی و با حضور در جلسات آموزشی تحت مداخلات آموزشی قرار گرفتند. برای جمع آوری اطلاعات از نماگر رشد حرکتی ویندرز و مقیاس رشد روانی- حرکتی گزل استفاده شد. داده‌های حاصل از پژوهش با استفاده از روش‌های آماری تحلیل کوواریانس مورد تجزیه و تحلیل قرار گرفت. یافته‌ها: با تحلیل داده‌های به‌دست آمده مشخص شد که عملکرد کلی فرزندان با نشانگان داون زیر ۵ سال، بین دو گروه مداخله و کنترل در مقیاس‌های رشد و تحول حرکتی، هم در زمینه رشد روانی- حرکتی و هم در زمینه مهارت‌های حرکتی درشت، تفاوت معنا‌داری دارد (P<0/001). نتیجه‌گیری: مداخلات به‌هنگام روان‌شناختی و آموزشی خانواده محور در والدین کودکان مبتلا به نشانگان داون، موجب افزایش رشد روانی – حرکتی و مهارتهای حرکتی درشت فرزندان آنها می‌شود.

Title: بررسی فرایندهای واجی کودکان فارسی زبان 2 تا ۴ ساله

http://rehabilitationj.uswr.ac.ir/article-1-308-fa.html

هدف: فرایندهای واجی، تغییراتی است که کودکان به هنگام بیان کلمات در نحوه تلفظ آن ایجاد می‌کنند تا بیان آن‌ها برای ایشان راحت‌تر شود. این پژوهش با هدف بررسی فرایندهای واجی کودکان عادی فارسی زبان ۲ تا ۴ ساله صورت گرفت روش بررسی: این پژوهش از نوع مقطعی و توصیفی-‌ تحلیلی است. آزمودنی‌های این پژوهش تعداد 128 دختر و پسر 24 تا ۴۸ ماهه تک زبانه فارسی زبان و فاقد هر نوع اختلال شدید شنوایی، بینایی، جسمانی، ذهنی، گفتار و زبان، عاطفی-روانی، ساختاری و کارکردی در تولید کننده‌های گفتاری هستند که به صورت تصادفی ساده از مهدکودک‌های مناطق مختلف شهر تهران انتخاب شدند. ابزار این پژوهش، آزمون محقق ساخته فرایندهای واجی، متشکل از 43 واژه یک تا چهار هجایی شامل تمام واج‌های زبان فارسی است که توسط آن فرایندهای واجی کودکان در مقوله‌های ساخت هجا، جانشینی همخوان، جانشینی واکه، همگونی کامل، جابجایی، و سایر فرایندها جمع آوری و بررسی می‌شود. داده‌های به‌دست آمده با استفاده از آزمون‌های آماری تی مستقل، من‌ویتنی، تحلیل واریانس یک‌طرفه و کروسکال والیس تحلیل شد یافته‌ها: در هیچ‌یک از فرایندهای واجی، تفاوت معناداری بین دخترها و پسرها مشاهده نشد (۰/۰۵>P)،اما فرایندهای واجی با افزایش سن روند رو به کاهش داشتند که این کاهش در فرایندهای ساخت هجا (۰/۰۰۱>P)، جانشینی همخوان (۰/۰۰۱>P)، جانشینی واکه (۰/۰۰3=P) و همگونی کامل (۰/۰۰3=P) معنادار بود. نتیجه‌گیری: نتایج این تحقیق حاکی از آن است که تفاوت جنسی تأثیری بر چگونگی فرایندهای واجی ندارد. اما با افزایش سن فرایندهای واجی رو به کاهش می‌روند که می‌تواند نشانگر رشد ادراکات واجی و تسلط بیشتر کودکان گروه‌های سنی بالاتر بر نحوه تلفظ لغات باشد.

Title: ارزیابی دینامیک حرکات ستون فقرات کمری در بیماران مبتلا به بی‌‌ثباتی قطعه‌ای کمری

http://rehabilitationj.uswr.ac.ir/article-1-307-fa.html

هدف: بی‌ثباتی قطعه‌ای ستون فقرات کمری یکی از زیرگروه‌‌های کمردردهای غیراختصاصی می‌باشد که هنوز علت و تشخیص قطعی آن با روش‌های موجود کاملاً روشن نیست. هدف از این مطالعه معرفی روشی جدید و تکرارپذیر است که قابلیت ارزیابی کینماتیک ستون فقرات کمری را در صفحه ساژیتال داشته باشد. روش بررسی: در این مطالعه که از نوع اعتبارسنجی و به روش مقایسه‌ای می‌باشد، 15 بیمار مشکوک به بی‌ثباتی قطعه‌ای ستون‌فقرات کمری که به صورت ساده و در دسترس انتخاب شده بودند و 15 فرد سالم که با بیماران جورشده بودند، تحت بررسی پارامترهای کینماتیک قطعات حرکتی کمری در حین حرکات فلکسیون و اکستانسیون با استفاده از فلوروسکوپی دیجیتال پالس قرار گرفتند. پارامترهایی همچون جابجایی انتقالی قطعه‌ای، جابجایی زاویه‌ای قطعه‌ای و طول خط مسیر مراکز چرخش لحظه‌ای (PICR) برای هر قطعه حرکتی محاسبه گردید. همچنین الگوی حرکتی فقرات کمری نیز بین دو گروه مورد مطالعه و مقایسه قرار گرفت. داده‌های حاصل با استفاده از آزمون‌های آماری مجذور خی، کولموگروف-اسمیرنوف، تی‌ مستقل و تحلیل‌واریانس با اندازه‌گیری‌های مکرر مورد تجزیه و تحلیل قرار گرفت. یافته‌ها: جابجایی انتقالی در حین حرکات فلکسیون و اکستانسیون در بیماران تفاوت معناداری را در قطعه حرکتی L5-S1 نشان داد (P<0/001). طول خط PICR نیز برای مهره‌‌های L1 و L5 در حین حرکت اکستانسیون در بیماران تفاوت معناداری را نشان داد (P<0/001). شش بیمار نیز الگوی حرکتی «ترتیبی-تأخیری» را نشان داده و تفاوت معناداری با سایرین داشتند (P<0/001). نتیجه‌گیری: در حالی‌که اغلب مطالعات گذشته نتوانسته‌‌اند تفاوت مشخصی بین بیماران دچار بی‌ثباتی قطعه‌ای مهره‌های کمری و افراد سالم گزارش نمایند، در این مطالعه تفاوتهایی بین این بیماران و افراد سالم در منطقه میانی دامنه حرکتی مشخص گردید. به‌نظر می‌رسد با استفاده از تجهیزات جدیدی همچون ویدئوفلوروسکوپی دیجیتال تشخیص مشکلاتی همچون ناپایداری قطعه‌ای مهره‌های کمری روشن تر شود.
